# 2D Nanostructures for Optoelectronic and Green Energy Devices

**DOI:** 10.3390/nano13061070

**Published:** 2023-03-16

**Authors:** Sangyeon Pak, Jung-Inn Sohn

**Affiliations:** 1 School of Electronic and Electrical Engineering, Hongik University, Seoul 04066, Republic of Korea; 2 Division of Physics and Semiconductor Science, Dongguk University-Seoul, Seoul 04620, Republic of Korea

Two-dimensional (2D) materials and nanostructures have gathered significant attention due to their excellent mechanical properties [1], unique electrical and optical characteristics [2,3], large surface-to-volume ratio, and chemical and environmental stability [4]. These features have led to the discovery of a large new family of 2D materials and a vast range of possible applications ranging from optoelectronics and electronics to energy conversion and saving applications. With the extensive catalogue of available 2D materials, ranging from metallic layers to semiconductors and insulators, their applications hold great promise for future innovative research in science and technology.

In recent years, many colleagues have been developing fundamental studies dedicated specifically to the design of 2D materials in optoelectronic and green energy devices due to their excellent opto-electrical and electrochemical performance. These research studies sit at the interface between engineering, material science, physics, and chemistry, and the importance of 2D materials and nanostructures in such applications calls for intensive experimental research assisted with engineering their fundamental and interface properties for enhancing the performance of various devices.

In this Special Issue “2D Nanostructures for Optoelectronic and Green Energy Devices”, we have collected nine high-quality, original research papers and one comprehensive review paper by outstanding scientists and engineers from relevant fields, covering the topics in optical properties and couplings in 2D nanostructures, [5,6,7,8] spectroscopic analysis in atomic scale, [9] 2D transistors, [10,11] 2D optoelectronics, [12] and 2D energy storage applications [13,14].

The synthesis and characterization of the new fundamental properties of 2D nanostructures are of fundamental importance in order to utilize the 2D nanostructures for optoelectronic and green energy device applications. Shin et al. [6] reported interlayer coupling effects in 2D heterostructures using low-frequency Raman spectroscopy, offering a route to observe the quality of the interface in 2D nanostructures. Singh et al. [7] and Pandey et al. [8] reported optical coupling in nanostructures and substrates, which can be used in nanophotonics and detection applications. Yoon et al. [9] reported the atomic arrangement of graphene-like ZnO examined using transmission electron spectroscopy. A-Rang Jang [11] reported graphene contact in a 2D transistor, which was effective in lowering the Schottky barrier in metal-2D semiconductor contact. Sangyeon Pak [10] reported a simple and effective fabrication route of p-type doping 2D MoS_2_ simply by spin coating CuCl_2_ molecules.

Two-dimensional nanostructures are also found extensively in the field of energy storage applications where nanomaterials can be used as the effective electrode materials with a large surface area in batteries and supercapacitors. The work by Wi and coworkers [13] reported layered graphite structures fabricated using the direct laser scribing of PI substrate, which is an effective electrode material in micro-supercapacitors when integrated with hydroquinone gel electrolyte. Such flexible micro-supercapacitors promise future energy storage components, especially in wearable applications. Liu et al. [14] utilized vanadium pentoxide nanofiber/carbon nanotube hybrid films as binder-free cathodes for zinc-ion batteries, achieving a high energy density and stable cyclability, demonstrating the promises for large-scale energy storage applications.

Last, the Special Issue includes a comprehensive review on the applications on 2D materials, perovskites, and 2D material/perovskite heterostructures for applications to optoelectronic devices, including solar cells and photodetectors, nicely summarized by Yuljae Cho and his co-workers [12]. Especially, the review summarizes various synthetic methods for 2D materials and perovskite materials, and the photodetector performance of various materials was adequately compared and summarized.

To summarize, this Special Issue is expected to attract and enrich readers through featuring all of the above-mentioned research articles and review articles. Especially, we express our sincere thanks to all the authors, reviewers, and editors that made a contribution to this Special Issue.

## Data Availability

Not Applicable.
